# Belladonna in Scarlatina

**Published:** 1844-09

**Authors:** 


*Belladonna in Scarlatina.*—It was not until some fifty cases of scarlatina had occurred in the Orphan House, of Charleston, S. C., and my father and I myself found ourselves burdened daily with new cases, and had reason to apprehend that every subject for the disease would be attacked by it, that I suggested a resort to the atropa belladonna. In accordance with my views, we proceeded to its administration to every child who had not yet contracted the disease, in the following manner: Five grains of the extract were dissolved in one ounce of cinnamon tea, and of this five drops were given morning and night to every child at or under 3 years of age, and one drop more for every year above that age.

In some few of the children under its influence, we observed a slight redness of the fauces and lips, with a faint efflorescence on the skin of the throat and cheeks, which might perhaps be attributed to a modified attack of the epidemic, or more probably to the effect of the remedy.

This practice was continued until the disappearance of the epidemic, in the course of eight weeks from the time of its first visitation, and three weeks from the commencement of the belladonna treatment. Not more than about 6 or 7 new cases occurred after the exhibition of this prophylactic, and the greater part of these occurred during the first week of its employment.

Such are the facts of the case, and it is to these facts I wish particularly to call attention. They are publicly sustained by a board of 12 commissioners or administrators, selected from among the most respectable citizens of Charleston, by the city council, as guardians of the "public's children," and of course cannot be controverted; indeed one of the members of the board, Mr. Daniel Ravenel, a gentleman of literary acquirements and high attainments, was himself instrumental in instituting the prophylactic remedy under discussion. Here, then, we have a large family, of 110 children, all ripe subjects for scarlatina. In the course of 5 weeks one half fall under its influence—a prophylactic is suggested—it is tried; and suddenly, in the midst of its malignancy and prevalence, the epidemic is arrested and soon disappears altogether.

*Dr. Logan, in N. O. Med. Jour.*

